# Colorimetric Sensors: Methods and Applications

**DOI:** 10.3390/s23249887

**Published:** 2023-12-18

**Authors:** Feng-Qing Yang, Liya Ge

**Affiliations:** 1 School of Chemistry and Chemical Engineering, Chongqing University, Chongqing 401331, China; 2 Nanyang Environment and Water Research Institute, Nanyang Technological University, Singapore 637141, Singapore

## 1. Introduction

Colorimetric sensors have attracted considerable attention in many sensing applications because of their specificity, high sensitivity, cost-effectiveness, ease of use, rapid analysis, simplicity of operation, and clear visibility to the naked eye [1]. A number of colorimetric sensors have been developed for the detection of metal and non-metal ions [2,3], proteins [4], small molecules [5,6], gases [7], viruses and bacteria [8,9], DNA/RNA [10,11,12], reactive oxygen species and acidity/base [13], as well as the biomarkers in clinical diagnostics [14,15,16]. However, the complex compositions of various samples and the low content of analytes make it critical to develop sensing tools with high sensitivity and selectivity. To meet the requirements of practical applications, colorimetric sensing technology and methods have been constantly developed and improved, for example, paper-based colorimetric sensors that can realize real-time on-site inspection, colorimetric detection sensors based on portable smartphones, colorimetric sensing arrays, dual-mode colorimetric sensors, and dual-response colorimetric sensors. Sixteen high-quality papers about biosensors and chemical sensors have been published in this Special Issue. One paper is a systematic review article, and fifteen papers are original research articles. These papers provide detailed studies in many areas, including the principles and mechanisms of colorimetric sensing; nanomaterials for colorimetric (bio)sensors for biomedical applications; paper-based colorimetric sensors; colorimetric sensors for cations, anions, and biomolecules; and colorimetric strips.

## 2. Overview of Contributions

In recent years, the emergence of advanced nanomaterials has greatly facilitated the development of colorimetric sensors. Recent advances in the design, fabrication, and application of colorimetric sensors were reviewed by Wu et al. (contribution 1). The advantages and application fields of colorimetric sensors were summarized, and the classification and sensing mechanisms of colorimetric sensors were systematically reviewed, including colorimetric sensors based on graphene and its derivatives, metal and metal oxide nanoparticles, DNA nanomaterials, quantum dots, and other materials. Finally, they discussed the future development trend of colorimetric sensors regarding the problems of stability, dispersibility, conductivity, and false-positive signals that still exist in these sensors.

Since abnormal levels of small biological molecules are associated with certain diseases, it is of great significance to develop colorimetric sensors that can detect these molecules quickly and sensitively. Based on curcumin-loaded polycaprolactone porous fiber mats with strong antioxidant activity, Kossyvaki et al. successfully established a reusable intelligent system for the rapid detection of alkaline vapors (volatile amine trimethylamine, and the biogenic amines cadaverine, putrescine, spermidine, and histamine) and/or antioxidant activity (contribution 2). Dopamine (DA) is an important neurotransmitter that plays a key role in neuropsychiatric disorders. Yang et al. developed a colorimetric sensing strategy for the detection of DA in human serum based on a rose-like CuS@Prussian blue/Pt composite with peroxidase-like activity (contribution 3). This colorimetric sensor exhibits excellent stability and performance, can quickly detect DA, and has the advantage of smartphone vision rapid inspection. Furthermore, Kang et al. designed a smartphone-assisted bedside detection platform for uric acid based on a photoexcited oxidase mimic, a two-dimensional (2D) imine-linked crystalline pyridine-based covalent organic framework (TpBpy COF) (contribution 4). This sensing platform enables the in situ detection of uric acid without special instruments with a limit of detection (LOD) of 3.1 µM and has been successfully applied in the determination of uric acid in human urine and serum samples. In addition, Furletov et al. reported a composite based on silver triangular nanosheets (AgTNPs) and polyurethane foam for the chemical analysis of organic thiols (contribution 5). It was found that the aggregation of AgTNPs under the action of thiols led to the reduction in and significant broadening of the local surface plasmon resonance band. The spectral changes occurring during the process can be recorded using a homemade color-recording device or even observed visually. This method is suitable for the analysis of pharmaceuticals and foods and demonstrates promising application potential in the solid-phase/colorimetric determination of organic thiols.

Wang et al. developed a novel peroxidase-like nanozyme nitro-functionalized metal–organic framework (MOF) NO_2_-MIL-53 (Cu) with high catalytic efficiency at neutral pH to overcome the dependence of peroxidase mimics on acidic pH (contribution 6). NO_2_-MIL-53 (Cu) exhibits excellent enzymatic mimetic activity, including peroxidase-, oxidase-, and laccase-like activity. Based on the peroxidase-like activity of NO_2_-MIL-53 (Cu), a colorimetric sensing platform for the detection of H_2_O_2_ and glucose was developed. The detection limits for H_2_O_2_ and glucose were 0.69 μM and 2.6 μM, respectively. Furthermore, to avoid the interference of moisture when detecting hydrogen peroxide vapor (HPV), Xie et al. developed a new dual-mode sensor for the detection of HPV (contribution 7). The sensor consists of a new composite material based on poly(3,4-ethylenedioxythiophene): polystyrene sulfonate (PEDOT: PSS) doped with ammonium titanyl oxalate (ATO). The material can be made into a thin film on an electrode substrate for chemical sensing of HPV. The adsorbed H_2_O_2_ will react with the ATO to cause a colorimetric reaction in the substance. PEDOT acts as a hydrophobic layer that protects the underlying sensor material from contact with moisture, thereby mitigating moisture interference during HPV detection. This dual-mode sensing method, which combines colorimetric and chemiresistive response, is not only more reliable but also improves the selectivity and sensitivity of detection.

Metal ions play a vital role in a variety of biological processes, nutrient cycling, and ecosystem function. However, imbalances in ion concentrations pose a potential risk to organisms and ecosystems, leading to health problems and environmental pollution. Three research articles in this Special Issue focus on the detection of metal ions. Pinto et al. reported a meso-triphenylamine BODIPY derivative for the highly selective detection of Cu^2+^ and Fe^3+^ (contribution 8). In the presence of Cu^2+^ and Fe^3+^, the compound shows a decrease in the absorption band at 305 nm and the appearance of new absorption peaks at 697 nm (Fe^3+^) and 700 nm (Cu^2+^), with a change in color from yellow to blue-green. Due to the strong complexation between the compound and Cu^2+^/Fe^3+^, the assay was highly sensitive, with an LOD of 0.63 µM and 1.06 µM, respectively. Furthermore, Alberti et al. developed a colorimetric paper-based analytical device (PAD) for the detection of Pd^2+^ in acidic aqueous solutions (contribution 9). PAD was produced by impregnating filter paper with an azo ligand, TazoC (2-(tetrazolylazo)-1,8 dihydroxy naphthalene-3,6-disulphonic acid), which exhibited high selectivity for detecting Pd^2+^ at low pH and could form complexes with Pd^2+^ and induce a color change. To enable rapid on-site detection of Ca^2+^ in biological samples, Tarara et al. developed a microanalytical paper-based device (μ-PAD) for the colorimetric determination of Ca^2+^ in saliva samples (contribution 10). Under alkaline conditions, Ca^2+^ complexed with Methylthymol Blue in μ-PAD, resulting in a color change on the surface of the device. The detection range was 30.71–84.15 mg/L, and LOD was 2.9 mg/L. This paper-based method provides a fast, low-cost, and portable solution for the determination of Ca^2+^ in saliva samples.

Shik et al. developed an optical sensor array for the identification of six model proteins and ten rennet samples (contribution 11). It was found that the protein products oxidized by sodium hypochlorite had certain oxidation properties and reacted with carbocyanine dyes IR-783 and Cy5.5-COOH to produce changes in color and fluorescence. Protein identification was achieved by processing the photographic image intensity of the 96-well plate with principal component analysis (PCA) and linear discriminant analysis (LDA).

To make the sample preparation technique more suitable for the concept of “green” analytical chemistry, Smirnova et al. developed a rapid extraction and colorimetric assay for synthetic food dyes based on an aqueous two-phase system (ATPS) based on a mixture of cationic and anionic surfactants (contribution 12). The method is based on the microextraction of ATPS followed by the spectrophotometric/colorimetric determination of food dyes. In addition, the colorimetric assays can be performed directly in the extract using a smartphone. This method is suitable for the determination of dyes in food samples and food processing industry wastewater. Phenolic compounds are organic pollutants that can be harmful to ecosystems, even at low concentrations. To detect and degrade phenolic compounds, Chai et al. reported a novel laccase mimic Tris(hydroxymethyl)aminomethane-Cu (Tris-Cu) (contribution 13). Tris-Cu nanozyme was prepared using a simple and rapid synthesis strategy based on the coordination of copper ions and amino groups in Tris. The nanozyme exhibited excellent catalytic activity against various phenolic compounds and can be used for the colorimetric detection of 2,4-dichlorophenol (2,4-DP) in the range of 10–400 μM. Tris-Cu also showed excellent removal of six phenolic compounds. Therefore, it shows broad application prospects in monitoring and degrading environmental pollutants. In addition, adenine phosphate-Cu (AP-Cu) nanozyme with multienzyme mimicking activity was prepared for the efficient degrading phenolic compounds and detection of hydrogen peroxide, epinephrine, and glutathione [17].

Ermakova et al. developed a dual-responsive and reusable optical sensor based on 2,3-diaminoquinoxaline for measuring the acidity of low-pH aqueous solutions (pH < 5) (contribution 14). They embedded hydrophilic quinoxaline into an agarose matrix to make pH-responsive polymers and paper test strips for visualizing semi-quantitative pH. Being exposed to acidic solutions with pH in the range of 1–5, the sensor can exhibit different color changes rapidly when the analysis is performed in daylight or under irradiation at 365 nm. In addition, they prepared pH indicators for quantitative analysis by immobilizing amphiphilic quinoxaline derivatives using Langmuir–Blodgett (LB) and Langmuir–Schäfer (LS) techniques. The resulting film has emission characteristics and excellent stability and can be used for dual-responsive pH measurements in the pH range of 1–3. The sensor demonstrates the potential to accurately measure pH in samples from complex environments.

To monitor the level of singlet oxygen (^1^O_2_) in biological media, Zanocco et al. developed an “off-on” fluorescent nanoprobe for ^1^O_2_ near-infrared multiphoton imaging (contribution 15). The nanoprobe consists of a naphthoxazole fluorescent unit and a singlet-oxygen-sensitive furan derivative attached to the surface of mesoporous silica nanoparticles. Under single-photon and multiphoton excitation, the nanoprobe reacts with singlet oxygen and enhances fluorescence up to 180-fold. Nanoprobes are easily internalized by macrophages and enable imaging of intracellular singlet oxygen under multiphoton excitation. In addition, Laman et al. designed a Miniaturized Opticallyvclear Thermal Enclosure (MOTE) system capable of convectively heating a sample while imaging it with transmitted light (contribution 16). The MOTE system can effectively perform paper-based Loop-Mediated Isothermal Amplification (LAMP) reactions for real-time detection of λ DNA, achieving sensitivity down to 10 copies/µL of the target concentration. This open-source, inexpensive, and low-power modular system provides a promising tool for different applications, such as molecular diagnostics, biochemical analysis, cell biology, and genomics.

In addition, many small molecules, including adenine phosphate, vitamin B3, and vitamin B6, were found to possess POD-like activity in our previous studies, which were successfully applied in the colorimetric detection of hydrogen peroxide, ascorbic acid, and glutathione, as well as total antioxidant capacity evaluation, respectively. Therefore, modifications of nanomaterials using these small molecules to increase their enzyme-like activity or endow them with multiple enzyme-like activity have great potential in improving the sensitivity and selectivity of related colorimetric detection methods [1].

## 3. Conclusions

This Special Issue focused on the principles and mechanisms of colorimetric sensors, fabricating methods, and related applications. To meet the requirements of practical applications, researchers have developed multifunctional colorimetric sensors, for example, based on the ATPS system for microextraction and colorimetric determination of food dyes, the titanium oxalate-doped PEDOT films as chemoresistive and colorimetric dual-mode sensors for the detection of hydrogen peroxide vapors, and optical sensor arrays for differentiating between protein and rennet samples. The combination of colorimetric sensors with smartphones, color-recording devices, colorimetric stripes, and optical sensing arrays not only improves the portability, sensitivity, and specificity of detection but also broadens the application field of colorimetric sensors. With the development of materials chemistry and analytical chemistry, it is believed that colorimetric sensors will make an even greater contribution to the chemical and biological sensing fields.
